# Based on the skeletal-muscle dual stem cell chemotaxis and bidirectional differentiation mechanism to reinterpret the “sinew-bone interdependence” theory

**DOI:** 10.3389/fcell.2026.1869812

**Published:** 2026-10-09

**Authors:** Jiahao Dong, Tao Chen

**Affiliations:** 1 The First Clinical Medical College, Yunnan University of Chinese Medicine, Kunming, Yunnan, China; 2 Yunnan Provincial Hospital of Traditional Chinese Medicine, Kunming, Yunnan, China

**Keywords:** bidirectional differentiation, bone marrow mesenchymal stem cells, sinew-bone interdependence, skeletal muscle satellite cells, stem cell chemotaxis, stem cell niche

## Abstract

The “sinew-bone interdependence” theory is a core paradigm in traditional Chinese medicine (TCM) orthopedics and traumatology. Rooted in the visceral manifestation doctrine of “the liver governing sinews and the kidney governing bones,” this theory emphasizes the structural dependency, functional synergy, and pathological inter-transmission between sinews and bones. However, traditional interpretive pathways have largely remained at the level of descriptive deduction concerning anatomy, visceral manifestations, and biomechanics, failing to elucidate the cellular executors and molecular mechanisms underpinning the sinew-bone association. In this theoretical article, we propose a hypothesis that intercellular communication between skeletal muscle satellite cells (MuSCs) and bone marrow mesenchymal stem cells (BMSCs) may provide a cellular-molecular basis for this classical theory. This article systematically reviews the molecular identity of MuSCs and BMSCs, the molecular basis of stem cell chemotaxis centered on the SDF-1/CXCR4 axis, and the existing evidence for the bidirectional chemotaxis of these two stem cell types. It further analyzes the network characteristics of the Wnt/β-catenin, BMP/Smad, and Notch signaling pathways in regulating lineage specification and the research progress on multi-target interventions by Chinese herbal medicines. Building on this foundation, we propose a testable hypothesis of “sinew injury recruiting bone, bone damage summoning muscle” and construct a “Dual Stem Cell-Chemotaxis-Differentiation” (DSCD) model. We emphasize that this model represents a theoretical framework requiring experimental validation rather than an established mechanism. This model redefines the sinew-bone system as a dynamic cellular niche maintained by a chemotactic axis, an exosome-based communication network, and a convergence of multiple signaling pathways. This article aims to propose measurable molecular connotations for classical theory, thereby stimulating further experimental investigation in TCM orthopedics in the era of regenerative medicine.

## 1 Introduction

The “sinew-bone interdependence” theory is one of the core paradigms in TCM orthopedics and traumatology. Its ideological origins can be traced back to the Huangdi Neijing (the Yellow Emperor’s Inner Classic). Grounded in the visceral manifestation doctrine that “the liver governs sinews, and the kidney governs bones,” it emphasizes the structural dependency, functional synergy, and pathological inter-transmission between sinews and bones. Traditional explanatory pathways primarily encompass three dimensions: anatomical association, functional synergy, and pathological inter-transformation (Jaladat et al., 2023; Kazemi et al., 2024). At the anatomical level, sinews (tendons, ligaments, fasciae) and bones form a mechanical coupling system through connective tissues such as joint capsules, a feature highly consistent with the microscopic damage linkage observed at the tendon-bone interface in modern musculoskeletal ultrasound (Qiang et al., 2024). At the functional level, the dynamic balance of “sinews serving the bones, bones providing a framework for the sinews” corresponds to the “muscle-bone co-activation” mechanism in modern biomechanics (Duan et al., 2024; Tan et al., 2024). At the pathological level, the correlation between clinical syndrome differentiation and imaging indicators such as synovial blood flow signals and joint effusion provides visual evidence for traditional pathogenesis like “kidney deficiency causing bone atrophy, liver blood failing to nourish sinews” (Qiang et al., 2024). The unique contribution of these explanatory pathways lies in integrating macroscopic symptoms with microscopic pathology, thereby forming a stepwise therapeutic strategy of “regulating sinews and setting bones — activating blood and unblocking collaterals — tonifying the liver and kidney” (Jaladat et al., 2023; Kazemi et al., 2024).

However, these pathways fundamentally remain descriptive deductions at the structural or functional level and have yet to answer a fundamental scientific question: precisely which cell types execute the “association” between sinews and bones, and through what molecular mechanisms is it achieved? In other words, traditional theory currently lacks an operational and verifiable cellular and molecular carrier.

Recent studies suggest potential clues to answering the above questions. Using lineage tracing and intravital imaging techniques, multiple studies have confirmed that skeletal muscle satellite cells (MuSCs) can breach tissue boundaries under specific microenvironmental stimuli, responding to chemotactic signals to migrate directionally toward distal injury sites. Their migration efficiency is regulated by a fibronectin splice variant (EDB^+^ FN) in the extracellular matrix (Lazure et al., 2023; Liu Y. et al., 2025). Furthermore, a youthful microenvironment can reverse the aberrant expression of approximately 50% of migration-related genes in aged MuSCs through epigenetic reprogramming, further indicating the decisive role of the microenvironment in stem cell chemotactic behavior (Lazure et al., 2023).

On the other hand, bone marrow/periosteal mesenchymal stem cells (BMSCs/PDCs) not only directly differentiate into myogenic cells during muscle injury repair but also activate satellite cells and inhibit inflammatory responses through the paracrine secretion of bioactive molecules like FGF and EGF, thereby accelerating muscle fiber regeneration (Ahmad et al., 2025; Lesinski et al., 2025; Marchand et al., 2025; Ding et al., 2026; Khaled et al., 2026; Salman et al., 2024; Enrick et al., 2023). Exosomes, serving as key mediators of intercellular communication, construct a bone-muscle signaling network by carrying molecules such as chemokines, cytokines, and nucleic acids, wherein the SDF-1/CXCR4 and HGF/c-Met pathways exhibit significant synergy (Yan et al., 2025; Zhou Z. et al., 2023). SDF-1 (CXCL12) not only mediates the directed homing of mesenchymal stem cells (MSCs) via the CXCR4 receptor but also activates downstream Wnt/β-catenin signaling to promote osteogenic differentiation and bone regeneration (Huang et al., 2025; Wang et al., 2024). In an osteoporosis model, SDF-1-overexpressing BMSCs significantly increased femoral bone mass by enhancing CXCR4-dependent homing efficiency and upregulated Wnt-1 expression to drive the transcription of osteogenic genes like RUNX2 and OSX (Liang et al., 2025; Zhong et al., 2024); this mechanism has also been validated in bone defect repair, manifested by paracrine acceleration of early mineralization and positive feedback loops maintaining microenvironmental homeostasis (Liu D. et al., 2025).

Notably, traditional Chinese medicine (TCM) compound formulas and monomeric components exhibit multi-target synergistic intervention characteristics in regulating stem cell chemotaxis and differentiation. The spleen-invigorating and kidney-tonifying formula improves the stem cell microenvironment by modulating the immune-inflammatory axis (e.g., inhibiting pro-inflammatory cytokines like TNF-α and IL-6), thereby enhancing the directed migration capacity of BMSCs (Sun et al., 2023; Ma et al., 2024). Total flavonoids of Drynaria fortunei (Gusuibu) can activate Wnt/β-catenin and OPG/RANKL/RANK signaling pathways, promoting BMSC osteogenic differentiation while inhibiting their adipogenic differentiation (Du et al., 2024; Qin et al., 2023). Icariin can upregulate the expression of chemokine receptor CXCR4, enhancing stem cell homing efficiency via the SDF-1/CXCR4 axis (Yang et al., 2025).

It is critical to note, however, that direct *in vivo* evidence for bidirectional chemotaxis between MuSCs and BMSCs remains limited. Much of the existing literature describes general mesenchymal stem cell homing or indirect muscle-bone crosstalk rather than definitive reciprocal migration between these two specific populations. Based on the aforementioned cellular and molecular biological advances, this paper proposes a hypothesis: a “dual stem cell chemotaxis and bidirectional differentiation” mechanism may exist between bone and muscle. In the pathological process of “sinew injury recruiting bone, bone damage summoning muscle,” MuSCs and BMSCs might achieve reciprocal chemotaxis and functional complementarity through remote dialogues mediated by chemokines and exosomes. Their bidirectional differentiation potential could be precisely regulated by microenvironmental signaling networks and TCM interventions. This paper will first review the main explanatory pathways of traditional theory, including their contributions and limitations; subsequently define the identities of bone-muscle dual stem cells, the molecular basis of their chemotaxis, and the evidence for reciprocal dialogue; then focus on the mechanism of lineage specification and research progress on TCM regulation; and finally construct a “dual stem cell—chemotaxis—differentiation” model to reinterpret the “sinew-bone interdependence” theory and explore its implications for TCM orthopedics and traumatology treatment principles and research paradigms. This theoretical reconstruction aims to propose measurable molecular connotations for classical doctrine and provide a theoretical framework for future experimental investigation. The core framework and operational logic of the theoretical model constructed in this paper are illustrated in Figure 1.

**FIGURE 1:**
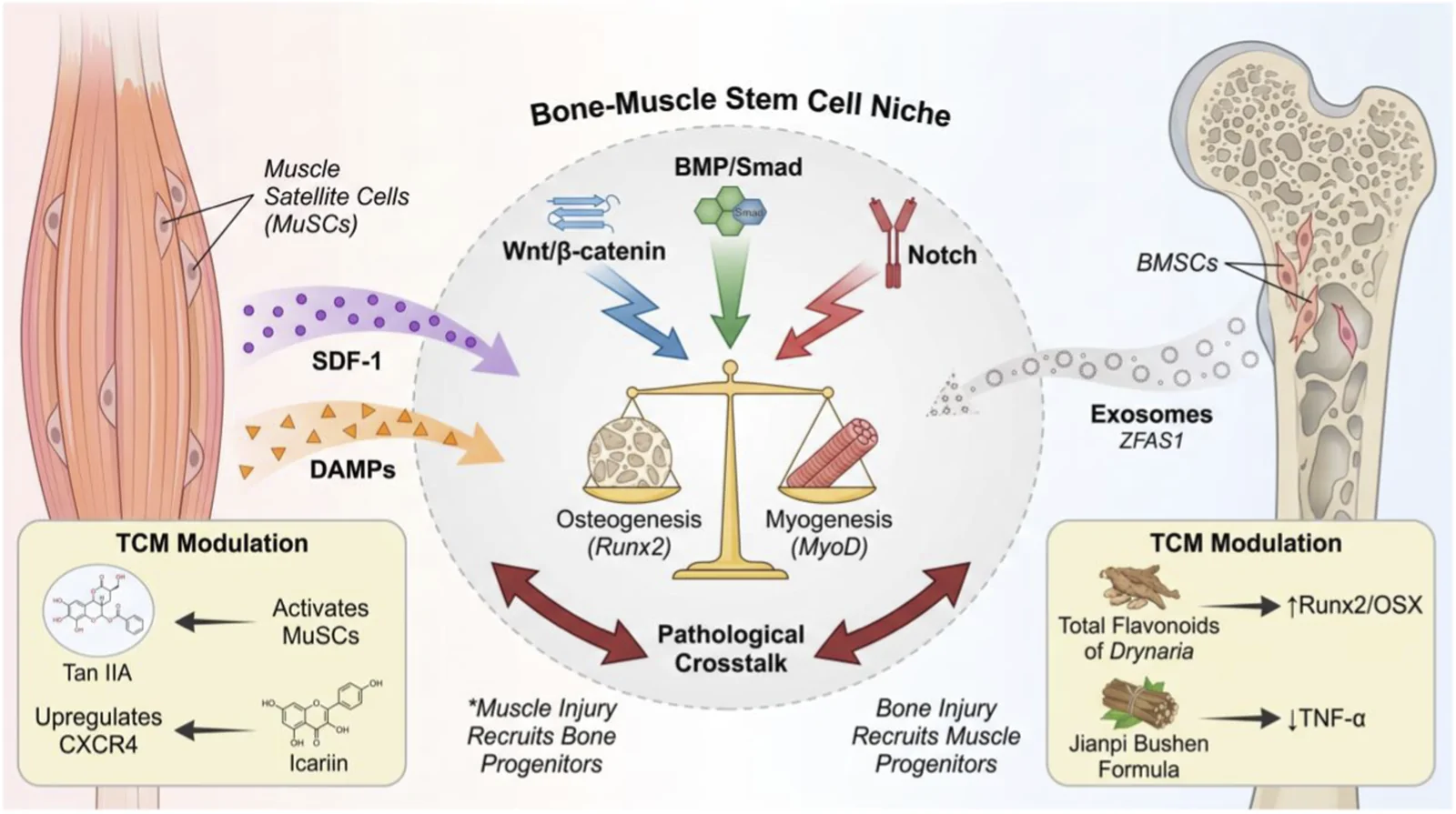
Reinterpretation model of the “sinew-bone interdependence” theory based on skeletal-muscle dual stem cell chemotaxis and bidirectional differentiation. This figure illustrates the core architecture of the DSCD model as proposed herein: Skeletal muscle satellite cells and bone marrow mesenchymal stem cells may hypothetically achieve bidirectional pathological inter-transmission—“sinew injury recruiting bone, bone damage summoning muscle”—through the SDF-1/CXCR4 chemotactic axis and exosome communication. Their lineage specification direction could be regulated by a Wnt/BMP/Notch signaling convergence network. Active components of traditional Chinese medicine (TCM) could theoretically target multiple nodes of this niche.

## 2 Connotation review of the traditional “sinew-bone interdependence” theory

### 2.1 Classical connotation: Structural dependency, functional synergy, and pathological inter-transmission

The structural dependency and functional synergy between sinews and bones represent foundational understandings within the theoretical system of TCM. These concepts are expounded upon throughout classics such as the Huangdi Neijing (The Yellow Emperor’s Inner Classic) and Yizong Jinjian (The Golden Mirror of Medicine), and have been continuously corroborated by modern anatomical and biomechanical research. At the anatomical level, sinews (encompassing dense connective tissues such as tendons, ligaments, and fasciae) and bones form direct mechanical couplings through attachment sites. For example, the anatomical association between the calcaneofibular ligament and the calcaneus (Zhang et al., 2023), and the coordinated positioning pattern of muscles and bony structures in the cranio-orbital region (Cheng et al., 2024), both characterize the role of this structural interlocking in ensuring musculoskeletal system stability. The Lingshu (Spiritual Pivot) chapter “Jingmai” (Meridians) summarizes the functional division of these three components as “bones serving as the framework, vessels as the nourishment, sinews as the rigidity”—bones providing the mechanical support framework, while sinews endow the system with dynamic tension. The functional complementarity between the two is echoed by modern research findings, such as the regulatory effect of skeletal muscle contraction on bone metabolism (Kirk et al., 2025; Ma et al., 2023) and the positive correlation between muscle strength and bone density (Kirk et al., 2023).

In the clinical practice of orthopedics and traumatology, “treating sinew and bone with equal emphasis” and “sinew and bone sharing disease” have become fundamental principles permeating diagnosis and treatment, their connotations fully embodying the structural-functional holism of the TCM perspective. Once “sinew” is defined as soft tissues like tendons, ligaments, and joint capsules, and “bone” as the skeletal framework, the pathological inter-transmission relationship between the two becomes clearly discernible: fractures are often associated with adjacent ligament injuries, such as ankle fractures accompanied by deltoid ligament rupture (Li M. et al., 2024; Spindler et al., 2024); ligament laxity or rupture can induce joint instability, thereby accelerating the progression of osteoarthritis (Meziani et al., 2023; Wen et al., 2025). This interactive injury pattern of mutual causality inversely corroborates the scientific value of “treating sinew and bone simultaneously.” For instance, in the treatment of ankle fractures in young adults, synchronously repairing ligaments and joint capsules alongside fracture reduction and fixation significantly improves functional prognosis, with an excellent-to-good rate on the AOFAS score reaching 98.3% (Shen et al., 2025), providing quantitative evidence-based support for the principle of “treating sinew and bone with equal emphasis.”

### 2.2 Existing interpretive pathways and their contributions and boundaries

As a core theoretical system in TCM orthopedics and traumatology, the explanatory pathways for “sinew-bone interdependence” have formed a multi-dimensional, systematic framework. Reviewing the existing literature, these can be summarized into three main pathways: the anatomical-functional correlation pathway, the meridian-visceral manifestation holistic pathway, and the mechanical-biological dynamic equilibrium pathway. Each pathway offers unique contributions to theoretical deepening and clinical application.

The anatomical-functional correlation pathway, centered on the concept of “sinews binding bones to facilitate joints,” emphasizes the morphological continuity and functional dependency between sinews and bones. Originating from the Suwen (Basic Questions) statement that “ancestral sinews govern binding bones and facilitating joints,” this pathway was systematized through the development by physicians during the Yuan and Ming dynasties. It views “bone as the framework, sinew as the rigidity” as the basic schema of mechanical support, while simultaneously pointing out that sinew flexibility is a necessary condition for maintaining joint range of motion (Wei et al., 2024). Its contributions lie in: first, clearly establishing “morphological structural changes in sinew and bone” as the fundamental pathological mechanism of disease, such as sinew dislodging from bone and sinew laxity causing bone weakness (Wei et al., 2024); second, directly guiding the formation of treatment principles like “regulating sinews and setting bones” and “reconnecting sinews and setting fractures,” providing theoretical grounding for manual reduction techniques [e.g., spinal balance adjustment and acupressure (Li JF. et al., 2024)]. Modern research has further corroborated the scientific nature of this pathway—the finding that biomechanical imbalance between sinew and bone in osteoporosis leads to deterioration of bone biomechanical properties pathologically confirms the therapeutic value of “nourishing bone through sinew” (Zheng et al., 2023).

The meridian-visceral manifestation holistic pathway relies on the theoretical framework of “visceral manifestations—meridians—five body constituents,” viewing sinews and bones as functional units linked to internal organs and meridians. The core viewpoint of this pathway is that the function of sinews and bones depends on visceral regulation by “the liver governing sinews, the kidney governing bones” and meridian nourishment through “the Taiyang governing sinews, the Shaoyang governing bones” (Wei et al., 2024; Wu et al., 2025); the onset of chronic sinew-bone diseases (e.g., chronic ankle instability, CAI) is closely related to “visceral deficiency and meridian qi disharmony,” necessitating holistic treatment through “regulating viscera to consolidate the root, unblocking meridians to activate collaterals” (Wu et al., 2025). Its innovation lies in integrating local sinew-bone pathology into the operational network of systemic qi, blood, yin, and yang—such as the clinical application of navel acupuncture to regulate the Kan and Li positions to restore the heart-kidney water-fire balance during cervical spondylosis treatment (Zhou et al., 2025). Contemporary “organ dialogue” research provides interdisciplinary resonance for this pathway; for example, the finding that the liver-gallbladder axis regulates sinew-bone metabolism via neuro-immune networks conceptually converges with the TCM holistic view of “the liver and gallbladder being interrelated” (Wang and Liao, 2024).

The mechanical-biological dynamic equilibrium pathway integrates traditional theory with modern biomechanics, proposing that maintaining sinew-bone homeostasis requires satisfying the mechanical adaptability condition of “combining motion and stillness.” The core contributions of this pathway manifest at two levels: first, it reveals the mechanobiological mechanisms of sinew-bone imbalance, such as abnormal bone remodeling due to insufficient mechanical loading in osteoporosis patients (Zheng et al., 2023); second, it drives innovations in rehabilitation techniques, as seen in exercise therapies like GLA:D Back, which enhance patients’ self-management capabilities to improve sinew-bone coordination in chronic low back pain (Heiberg et al., 2023), representing a clinical translation of this pathway. Modern musculoskeletal pain research further indicates that abnormal central nervous system regulation of muscle tone can trigger sinew-bone dysregulation, requiring integrative interventions like the Feldenkrais method to re-establish the dynamic neuro-musculo-skeletal balance (Giorgi et al., 2023; Cacciatore et al., 2024), thereby extending the explanatory boundaries of this pathway from the dimension of neural regulation. The main contributions and boundaries of these three interpretive pathways are summarized in Table 1.

**TABLE 1:** Summary of key experimental evidence for skeletal-muscle dual stem cell chemotaxis and crosstalk.

Research direction	Model/Source	Core findings	Pathways/Molecules involved	Evidence level
Microenvironmental regulation of MuSCs	Mouse skeletal muscle stem cells	Young niche reverses aberrant expression of ∼50% of migration-related genes in aged MuSCs via epigenetic reprogramming	EDB^+^ FN, epigenetic reprogramming	Direct
Trans-tissue migration of MuSCs	Mouse injury models	MuSCs can breach tissue boundaries and migrate directionally toward distal injury sites in response to chemotactic signals	SDF-1/CXCR4, EDB^+^ FN	Direct
BMSC homing to muscle injury site	Muscle injury mouse model	Local SDF-1α injection enhances ASC migration to ischemic muscle, promoting angiogenesis and myofiber realignment	SDF-1α/CXCR4	Indirect (ASCs)
Paracrine effects of BMSCs on muscle repair	Muscle injury animal models	BMSCs activate satellite cells and inhibit inflammation via paracrine FGF, EGF, accelerating muscle fiber regeneration	FGF, EGF, paracrine signaling	Direct
SDF-1/CXCR4 axis in bone regeneration	Osteoporotic mouse model	SDF-1-overexpressing BMSCs increase bone mass by enhancing CXCR4-dependent homing and upregulating wnt-1-driven osteogenic genes	SDF-1/CXCR4, wnt/β-catenin, RUNX2, OSX	Direct
TCM monomer regulating stem cell fate	*In vitro* BMSC culture	Total flavonoids of drynaria fortunei activate wnt/β-catenin and OPG/RANKL pathways, promoting osteogenesis and inhibiting adipogenesis	Wnt/β-catenin, OPG/RANKL/RANK	Direct (*in vitro*)
TCM monomer regulating chemotaxis efficiency	Stem cell transplantation model	Icariin upregulates CXCR4 expression, enhancing homing efficiency via SDF-1/CXCR4 axis	SDF-1/CXCR4	Direct
Exosome-mediated bone-muscle crosstalk	Co-culture/*in vivo* models	Exosomes carrying chemokines, cytokines, and nucleic acids construct a bone-muscle signaling network	SDF-1/CXCR4, HGF/c-met, exosomes	Emerging
Central nervous system regulating bone metabolism	Bone injury mouse model	Neural stem cell-derived exosomes containing PLIN5 protein promote bone regeneration by regulating local lipid homeostasis	PLIN5, exosomes	Direct
Sensory nerve regulating BMSC differentiation	*In vitro* BMSC culture	CGRP from peripheral nerve endings enhances BMSC osteogenic differentiation via p38 MAPK and wnt6/β-catenin	CGRP, p38 MAPK, wnt6/β-catenin	Direct
Direct evidence for dual stem cell bidirectional chemotaxis (gap)	N/A	No lineage-tracing evidence currently exists for *in vivo* bidirectional migration between endogenous MuSCs and BMSCs	N/A	Critical evidence gap

## 3 Skeletal-muscle dual stem cells: identity definition, chemotactic basis, and reciprocal dialogue

### 3.1 Molecular definition of bone-related and muscle-related stem cells

Development and regeneration in the skeletal and muscular systems depend on two distinct stem cell populations with differing molecular signatures and functional specializations. Bone-related stem cells are primarily represented by bone marrow mesenchymal stem cells (BMSCs), while muscle-related stem cells are typified by skeletal muscle satellite cells (MuSCs). Although both originate from the mesoderm and exhibit overlapping differentiation potential and microenvironmental responsiveness, their molecular regulatory networks differ significantly. BMSCs possess tri-lineage differentiation potential into osteogenic, adipogenic, and chondrogenic lineages; their fate decision toward the osteogenic direction is strictly controlled by the Wnt/β-catenin signaling pathway—the secreted antagonist SFRP1 acts as a negative regulatory node by blocking the binding of Wnt ligands to FZD receptors (Yu et al., 2025; Nookaew et al., 2024). MuSCs rely on the temporally sequential expression of myogenic regulatory factors (MRFs) like MYOD and MYOG to drive muscle fiber formation and repair. Notably, these two populations are not isolated lineages: miR-206-3p can simultaneously regulate myogenesis and osteogenic differentiation by targeting SFRP1, suggesting a potential basis for bone-muscle co-regulation (Yu et al., 2025; Nookaew et al., 2024). This finding suggests that, in specific developmental and injury repair contexts, the two stem cell types may share some signaling interfaces, providing a potential molecular prerequisite for the chemotactic crosstalk and lineage plasticity discussed below.

### 3.2 Molecular basis and multi-level regulatory network of stem cell chemotaxis

Stem cell chemotaxis (homing) is a core cellular behavior for maintaining tissue homeostasis and initiating injury repair, essentially constituting a multi-step, multi-level dynamic interaction process between stem cells and their microenvironment (niche) (Poirier, 2025). The key to initiating chemotaxis lies in specific ligand-receptor recognition: the binding of the chemokine CXCL12 (also known as SDF-1) to its receptor CXCR4 activates Rho GTPase family members (such as Rac1, Cdc42) through G-protein-coupled signaling, driving actin polymerization and directed cell migration (Liu et al., 2026; Fiore et al., 2025). A recent study revealed a novel regulatory mode at the epigenetic level of chemotactic capacity: the epigenetic regulator SP140 negatively regulates hematopoietic stem cell homing ability by restricting the chromatin accessibility of key endothelial-to-hematopoietic transition genes (RUNX1, GATA2, *etc.*); transient inhibition of SP140 significantly elevates homing efficiency, providing an actionable strategy for the *ex vivo* expansion of functional stem cells (Liu et al., 2026). Moreover, integrin-mediated cell-matrix adhesion converts mechanical signals into chemotactic commands through the YAP/TAZ pathway, indicating that mechanosensation is also an indispensable regulatory dimension for the spatial localization of stem cells (Fiore et al., 2025; Elosegui-Artola et al., 2017). Force applied to the nucleus directly drives YAP nuclear translocation by decreasing the mechanical restriction of nuclear pores, providing a mechanistic link between mechanotransduction and stem cell chemotaxis (Elosegui-Artola et al., 2017).

At the overall regulatory network level, stem cell chemotaxis exhibits multi-layered integration of the immune microenvironment, metabolic status, and cellular subpopulation heterogeneity. Cytokines within the immune microenvironment can dynamically modulate chemotactic sensitivity: TNF-α enhances the recruitment efficiency of mesenchymal stem cells (MSCs) to inflammatory injury sites by upregulating CCR5 surface expression (Poirier, 2025; von Maltzahn, 2026). Metabolic reprogramming is also deeply involved—hypoxia-inducible factor HIF-1α strengthens the tropism of stem cells to low-oxygen microenvironments by activating the glycolytic pathway, a mechanism particularly prominent in the homing behavior of stem cells within the tumor microenvironment (von Maltzahn, 2026; Khasawneh and Abu-El-Rub, 2022). Severe hypoxia has been shown to disrupt SDF-1 and CXCR4 expression in MSCs, thereby impairing their adhesion and migration capacities (Khasawneh and Abu-El-Rub, 2022). Single-cell transcriptomic analysis further reveals the molecular basis of chemotactic heterogeneity: excitatory neuronal precursors highly express the Semaphorin 3A receptor Neuropilin-1, whereas inhibitory neuronal precursors depend on the Ephrin-Eph bidirectional signaling system. This molecular subtyping based on receptor expression profiles provides a precise entry point for targeted regulation of the migratory behavior of specific stem cell subpopulations (Liu et al., 2026; Elosegui-Artola et al., 2017).

### 3.3 Evidence of dual stem cell reciprocal chemotaxis and the interface molecular basis

We must emphasize that direct evidence for bidirectional chemotaxis between MuSCs and BMSCs remains limited. Nevertheless, mounting evidence suggests that skeletal muscle and the skeletal system do not operate independently during injury repair but may achieve a coordinated response through reciprocal chemotaxis between their respective stem cells. MuSCs and BMSCs may build a bidirectional communication network by secreting chemokines and extracellular matrix (ECM) components, jointly orchestrating the injury repair program (Dong et al., 2024; Kong et al., 2024). Damage-associated molecular patterns (DAMPs) released following skeletal muscle injury exert a dual mobilization effect: they activate local MuSC proliferation and differentiation while also potentially transmitting signals distally to promote the migration of BMSCs to the muscle injury site (Author anonymous, 2024; Huang et al., 2023). The molecular basis of this chemotactic action primarily relies on the SDF-1α/CXCR4 axis—local injection of SDF-1α significantly enhances the migration efficiency of adipose-derived stem cells (ASCs) to ischemic muscle tissue, simultaneously promoting angiogenesis and the re-alignment of muscle fibers, indicating that this chemotactic axis possesses dual functions of cell mobilization and tissue remodeling in ischemic muscle injury repair (Huang et al., 2023). Furthermore, single-cell transcriptomic analysis has identified the co-expression of the ROBO4 receptor by myogenic progenitor cells and osteogenic precursor cells; this molecule participates in cell migration regulation and may play an interface-specific role in the regeneration of muscle-bone interface tissues (Li H. et al., 2024). However, these studies do not directly demonstrate *in vivo* bidirectional migration between endogenous MuSCs and BMSCs. This represents a critical gap that future lineage tracing studies must address. The cellular behaviors and key molecular basis of this hypothesized reciprocal chemotaxis are summarized in Figure 2.

**FIGURE 2:**
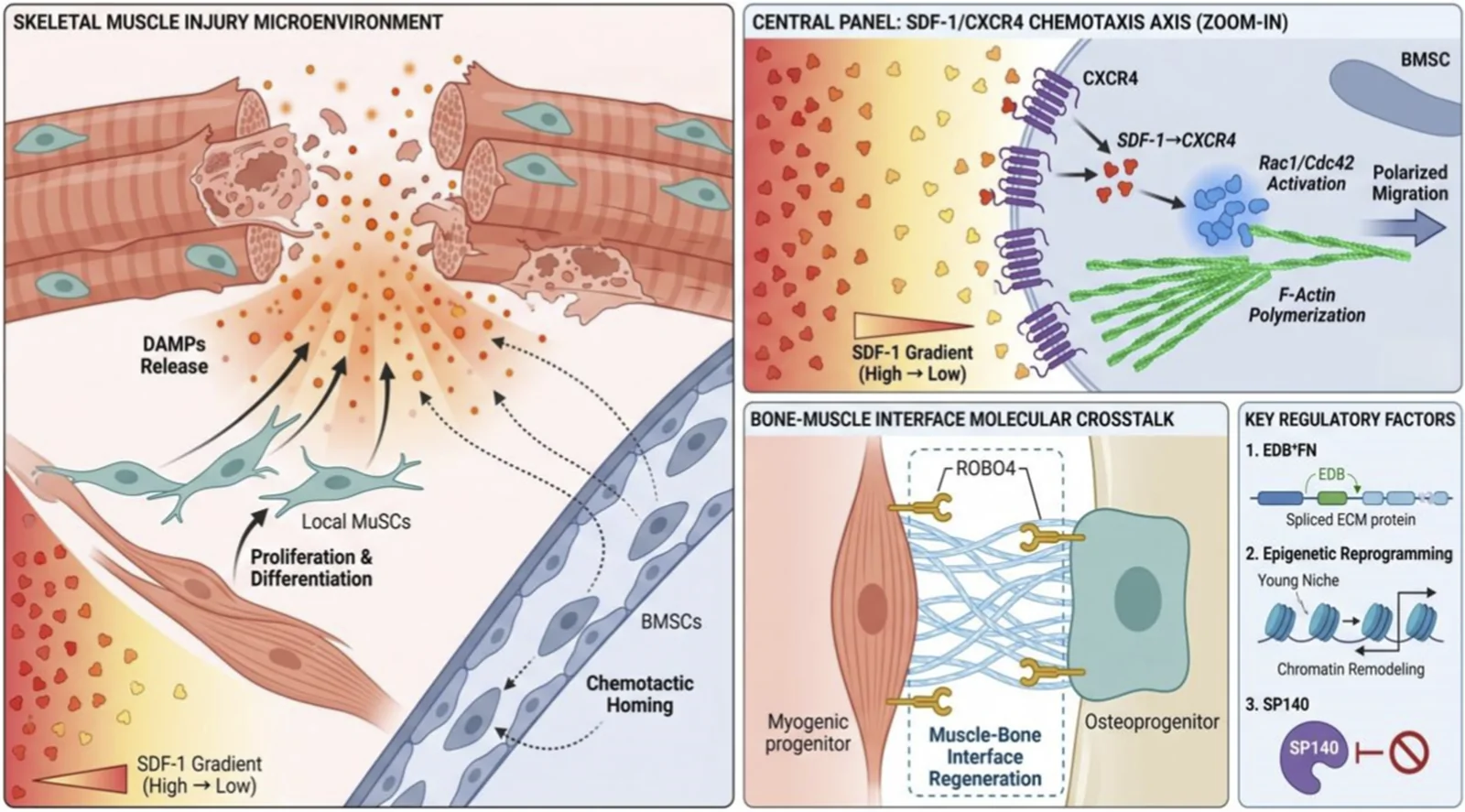
Schematic diagram of hypothesized reciprocal chemotaxis and interface molecular basis between skeletal muscle satellite cells and bone marrow mesenchymal stem cells. This figure represents a theoretical hypothesis requiring experimental validation. DAMPs released following skeletal muscle injury are hypothesized to exert a dual function, locally activating MuSCs and distally mobilizing BMSCs; the SDF-1/CXCR4 axis may drive the directed migration of BMSCs along the chemokine concentration gradient by activating Rac1/Cdc42; the ROBO4 receptor, co-expressed by myogenic progenitors and osteogenic precursors, may participate in regeneration regulation at the muscle-bone interface tissue. EDB^+^ FN, fibronectin splice variant containing extra domain B; SP140, epigenetic regulator.

## 4 Bidirectional differentiation of stem cells: from lineage restriction to cross-tissue repair potential

### 4.1 Conceptual definition of “bidirectional differentiation” and its bone-muscle extension

Stem cell bidirectional differentiation refers to the capacity of a unique pluripotent stem cell population to differentiate simultaneously or sequentially into two distinct lineage cells. This concept has recently gained clear experimental definition through the establishment of novel culture systems. While conventional pluripotent stem cells (such as naïve or primed states) typically exhibit a propensity to differentiate into the three embryonic germ layers, the breakthrough feature of bidirectional pluripotent stem cells (BPSCs) is their simultaneous expression of the pluripotency marker OCT4 and the trophoblast marker CDX2, thereby crossing the early boundary between embryonic and extra-embryonic lineages. This bidirectional potential is not a random event but is achieved through molecular reprogramming triggered by the hyperactivation of the Wnt signaling pathway, wherein Lef1-mediated epigenetic remodeling plays a pivotal role, allowing cells to bypass conventional lineage restriction (Liu K. et al., 2025). Extending this concept to the bone-muscle system, it is theoretically plausible to infer that if stem cells derived from bone and muscle sources can, under specific microenvironmental conditions, breach their inherent lineage constraints and exhibit cross-tissue differentiation potential, the cellular mechanism of “sinew-bone mutual nourishment” could gain a fundamental explanation. The subsequent review of core signaling pathways serves precisely to provide a molecular framework for this extension.

### 4.2 Core signaling pathways and epigenetic modification networks regulating bidirectional differentiation

The lineage specification capacity of stem cells is governed by a sophisticated network composed of multiple signaling pathways that maintain the transition between stem cell self-renewal and lineage commitment through dynamic equilibrium. The Wnt/β-catenin, BMP/Smad, and Notch signaling pathways play central roles, with their synergistic or antagonistic interactions collectively forming the molecular basis for bidirectional differentiation (Weatherbee et al., 2023; Hergenreder et al., 2024). Wnt signaling activation maintains the pluripotent state by stabilizing β-catenin, while its inhibition drives neuroectodermal differentiation. This bidirectional regulatory characteristic is particularly prominent in human induced pluripotent stem cell (iPSC) models (Hergenreder et al., 2024; Ewoldt et al., 2025). BMP signaling promotes mesendodermal differentiation through the Smad1/5/8 phosphorylation cascade while simultaneously inhibiting neuroectodermal fate; the crosstalk between BMP and Wnt pathways forms a “signal threshold window,” where the choice of differentiation direction depends, to a considerable extent, on the dynamic balance of signal intensities within this window (Weatherbee et al., 2023; Xu et al., 2024). The lateral inhibition mechanism of Notch signaling maintains stem cell population heterogeneity through Hes/Her family transcription factors, with its dynamic oscillation frequency encoding distinct differentiation commands (Mosbacher et al., 2023; Soma et al., 2024).

Epigenetic modifications form feedback loops with the aforementioned signaling pathways, further enriching the dimensions of bidirectional regulation. In iPSC differentiation models, inhibition of the histone demethylase LSD1 significantly accelerates cortical neuron maturation, suggesting a deep coupling between the epigenetic clock and differentiation signals (Hergenreder et al., 2024). Similarly, deletion of DNA methyltransferase DNMT3A leads to differentiation arrest in trophoblast stem cells, confirming that synergy between the epigenetic layer and BMP signaling is a necessary condition for determining lineage specificity (Weatherbee et al., 2023). The corresponding activation patterns of these pathways in the bone-muscle system—such as Wnt favoring osteogenesis, BMP participating in myogenic precursor regulation, and Notch maintaining muscle satellite cell quiescence—provide a directly translatable signaling framework for understanding how BMSCs and MuSCs might be differentially or synergistically guided to differentiate within the same injury microenvironment.

### 4.3 Multi-target regulatory characteristics of traditional chinese medicine intervention in stem cell bidirectional differentiation

The regulatability of stem cell lineage specification capability makes it an important intervention target in tissue repair and regenerative medicine. Traditional Chinese medicine (TCM), as a natural intervention modality with multi-component, multi-target characteristics, has recently shown unique advantages in precisely regulating stem cell fate decisions. Studies indicate that TCM can influence the differentiation balance of stem cells between lineages such as osteogenic/adipogenic and neuronal/glial lineages by modulating key signaling pathways, metabolic reprogramming, and microenvironmental remodeling, providing new strategies for treating degenerative diseases, metabolic disorders, and tissue injuries (Du et al., 2024; Sui et al., 2024; Zhou C. et al., 2023). The emerging concept of muscle-bone stem cell exchange highlights the potential for targeting resident mesenchymal stromal cells as a therapeutic strategy for musculoskeletal disorders (Sui et al., 2024).

In the field of bone metabolism, active components of TCM, such as flavonoids (e.g., Icariin, Baicalein) and saponins (e.g., Ginsenoside Rg1, Morroniside), regulate the osteogenic-adipogenic differentiation balance of BMSCs through pathways like Wnt/β-catenin and BMP-SMAD. For instance, Morinda officinalis saponins (MOS) promote osteogenic differentiation of human umbilical cord mesenchymal stem cells (HUC-MSCs) by activating BMP4-SMAD signaling while inhibiting PPAR-γ expression to reduce lipid accumulation (Zhou et al., 2024; Liu et al., 2024). Icariin reverses ketogenic diet-induced bone loss by inhibiting mTOR phosphorylation and upregulates osteogenic markers such as ALP and OCN (Liu et al., 2024). These findings reveal molecular mechanisms by which TCM influences stem cell fate decisions through epigenetic modification and metabolic regulation (e.g., LDHA-mediated lactate metabolism) (Sui et al., 2024; Zhou C. et al., 2023). In muscle tissue repair, Tanshinone IIA can promote the proliferation and differentiation of muscle stem cells (MuSCs) by activating the MAPK/Akt signaling pathway, thereby accelerating the regeneration and functional recovery of injured muscle (Liu et al., 2023). The intersection of these signaling pathways and the molecular network of multi-target TCM regulation are illustrated in Figure 3.

**FIGURE 3:**
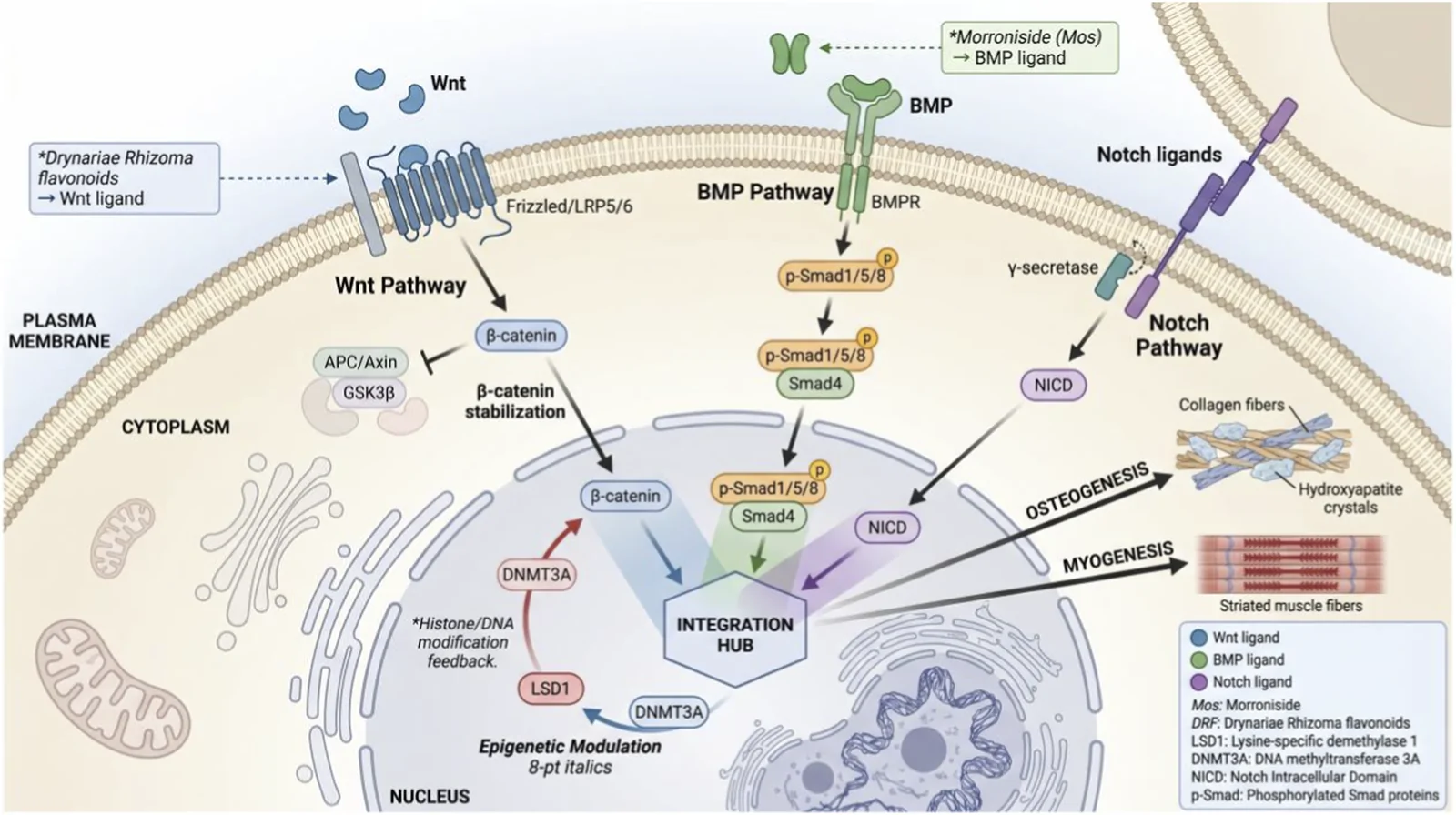
Schematic diagram of the intersection of wnt/β-catenin, BMP/Smad, and notch signaling pathways regulating the bidirectional differentiation of bone-muscle stem cells and multi-target intervention by traditional Chinese medicine. Three signaling pathways transduce intracellularly and converge within the nucleus, jointly regulating the transcription of osteogenesis-related genes and myogenesis-related genes. Active components of traditional Chinese medicine (TCM) can perform multi-nodal interventions at the ligand-receptor level, intracellular signal transduction level, and epigenetic level, embodying the characteristic of TCM in achieving multi-target regulation of the stem cell niche.

## 5 Cellular-molecular reinterpretation of the “sinew-bone interdependence” theory: construction of the DSCD model

### 5.1 Core hypothesis: the cellular-molecular basis of “sinew injury recruiting bone, bone damage summoning muscle”

Based on the aforementioned evidence of stem cell chemotaxis and lineage plasticity, this paper proposes a testable hypothesis for the “sinew-bone interdependence” theory: In the pathological process of “sinew injury recruiting bone, bone damage summoning muscle,” skeletal muscle satellite cells (MuSCs) and bone marrow mesenchymal stem cells (BMSCs) may achieve bidirectional chemotaxis and functional complementarity through remote dialogues mediated by chemokines and exosomes, potentially forming the cellular execution basis for sinew-bone mutual nourishment.

The fate decision of BMSCs—the balance between osteogenic and adipogenic differentiation—is jointly controlled by local microenvironmental signals (RANKL/OPG axis, Wnt/β-catenin pathway) and systemic factors (neuroendocrine regulation) (Kim et al., 2024; Zaidi et al., 2023). BMSCs can deliver exosomal lncRNA-ZFAS1, which competitively binds miR-9 to relieve its inhibition of IGF-1, thereby promoting osteoblast proliferation and differentiation (Sun et al., 2025); conversely, the loss of the aging-associated splicing factor YBX1 leads to aberrant splicing of osteogenic genes like Fn1 and Spp1 in BMSCs, resulting in osteoporosis (Xiao et al., 2023). These findings indicate that the functional complementarity of stem cells depends not only on cell-autonomous properties but is also controlled by a sophisticated intercellular communication network.

The regulation of chemotactic behavior also displays multimodal characteristics. Calcitonin gene-related peptide (CGRP) secreted by peripheral nerve endings can enhance the osteogenic differentiation capacity of BMSCs via the p38 MAPK and Wnt6/β-catenin pathways (Wu et al., 2023; Kong et al., 2023); neural stem cells (NSCs) from the central nervous system deliver exosomes containing PLIN5 protein to bone injury sites, promoting bone regeneration by regulating local lipid metabolism homeostasis (Kong et al., 2023). This bidirectional regulatory model of the neural-skeletal axis may corroborate, at the cellular-molecular level, the holistic view in TCM that “sinews and bones are connected with the internal organs”—the sinew-bone system does not operate in isolation but is deeply integrated with systemic regulatory systems through stem cell communication networks.

### 5.2 Architecture of the DSCD model: dual stem cell modules, chemotactic dialogue, and differentiation regulation

Based on the above evidence, this paper constructs a “Dual Stem Cell-Chemotaxis-Differentiation” (DSCD) model to systematically hypothesize the cellular mechanisms of sinew-bone interaction. The core architecture of this model encompasses three functional modules: (1) Dual Stem Cell Module—BMSCs and MuSCs serve as representative stem cell populations of bone and muscle, respectively carrying osteogenic/adipogenic/chondrogenic and myogenic differentiation programs; (2) Chemotactic Dialogue Module—centered on the SDF-1/CXCR4 axis as the core chemotactic pathway and employing exosomes as long-distance signal carriers, this module is proposed to achieve the bidirectional recruitment of the two stem cell types within the injury microenvironment; (3) Differentiation Regulation Module—the signaling network formed by the convergence of Wnt/β-catenin, BMP/Smad, and Notch signaling pathways may differentially guide stem cells toward osteogenic or myogenic differentiation, depending on the nature of the injury (bone injury or sinew injury) and the intensity of microenvironmental signals.

The key innovation of the DSCD model lies in transcending the conventional thinking framework of single-lineage repair. By simulating the dynamic intercellular interaction networks observed during development (such as the lateral induction and inhibition modes mediated by Notch signaling (Woo et al., 2025; Sullivan et al., 2023)), it constructs a spatiotemporally specific “stem cell niche.” In the context of bone-muscle interface regeneration, this model, through the precise regulation of the synergistic actions involving the TGF-β/GSK-3/ECM signaling axis (Lei et al., 2023) and the c-Kit/PI3K/Akt pathway (Xu et al., 2025), could both drive vascularization during endochondral ossification (He et al., 2023) and guide the directional alignment of muscle fibers, thereby achieving the functional integration of bone and muscle within a biomimetic microenvironment (Woo et al., 2025). This paradigm shift from single-tissue repair to bone-muscle composite unit regeneration provides an operational theoretical framework for understanding the cellular mechanism of “treating sinew and bone with equal emphasis.”

### 5.3 Complementary relationship between new and old interpretations and the theoretical advancement of the DSCD model

The contribution of the traditional interpretive pathways lies in their top-down deductive approach, establishing the macroscopic plausibility of “sinew-bone interdependence” from three dimensions: anatomy, visceral manifestations, and biomechanics. However, all three pathways fail to address the cellular executors and molecular means of sinew-bone interaction. This model intervenes precisely at this gap, with its core advancement lying in the shift from “phenomenological description” to hypothesized mechanistic deconstruction: proposing the bidirectional chemotaxis and differentiation of MuSCs and BMSCs as potential cellular carriers of sinew-bone mutual nourishment, thereby filling traditional theory with trackable molecular trajectories. If the traditional pathways delineate the “what” and “why” of the sinew-bone system, this model begins to answer the “how.” The specific differences and complementary relationships between the two in multiple dimensions are concisely presented in a juxtaposed format in Table 2.

**TABLE 2:** Multi-dimensional comparison between traditional interpretive pathways of the “sinew-bone interdependence” theory and the newly constructed DSCD model.

Comparison dimension	Traditional interpretive pathways	DSCD model (constructed here)
Core paradigm	Visceral manifestations – Meridians – Biomechanics	Dual stem cell niche
System definition	Mechanical coupling device, meridian conduction link, biomechanical equilibrium entity (macroscopic functional interface)	Dynamic cellular interface maintained by SDF-1/CXCR4 chemotactic axis, exosome communication network, and Wnt/BMP/Notch signal convergence
Core executor	Undifferentiated macroscopic “sinew” and “bone”	Synergistic action of MuSCs and BMSCs
Pathological inter-transmission mechanism	Clinical descriptive deduction based on anatomical association and functional imbalance	Sinew injury recruiting bone: MuSCs mobilize BMSC homing via DAMPs/SDF-1; “bone damage summoning muscle”: BMSCs activate MuSCs via exosomes
Explanation of bidirectional differentiation	Functional regulation by “liver governing sinews, kidney governing bones” (visceral-meridian nourishment)	Direct regulation by the intersecting Wnt/BMP/Notch signaling network + epigenetic feedback + exosome-mediated exchange of differentiation commands
Scientific connotation of treatment principles	Tonifying liver and kidney, strengthening sinews and bones (visceral regulation)	Multi-nodal intervention targeting the stem cell niche (modulating chemotactic intensity, correcting differentiation deviation, remodeling microenvironmental homeostasis)
Evidence-based methods	Clinical observation, imaging correlation, mechanical testing (revealing correlations)	Lineage tracing, single-cell multi-omics, conditional gene knockout, exosome tracking (establishing causality)
Interface with cutting-edge disciplines	Conceptual-level resonance	Deep integration with cutting-edge paradigms of “bone-muscle axis” and “organ dialogue”

Viewed from the system boundary perspective, traditional pathways define the sinew-bone system as a mechanical coupling device, a meridian conduction link, or a biomechanical equilibrium entity—essentially all macroscopic functional interfaces. This model redefines the sinew-bone system as a “dual stem cell niche”—a dynamic cellular interface maintained by the SDF-1/CXCR4 chemotactic axis, an exosome communication network, and the convergence of Wnt/BMP/Notch signaling. This redefinition transforms the sinew-bone system from a structural unit passively receiving regulation into an active cellular ecosystem with intrinsic repair and self-maintenance capabilities, achieving conceptual alignment with contemporary cutting-edge research paradigms like the “bone-muscle axis” and “organ dialogue.”

From the perspective of evidence-based methodology, traditional pathways rely on clinical observation, imaging correlation, and mechanical testing—tools that can reveal correlations but struggle to establish causality. The technologies underpinning this model, such as lineage tracing, single-cell transcriptomics, conditional gene knockout, and exosome tracking, endow the hypothesis of “sinew injury recruiting bone, bone damage summoning muscle” with scientifically testable and falsifiable attributes. The falsifiability of a theory is a key marker of its transition from empirical induction to a scientific hypothesis.

In the therapeutic dimension, the “regulating sinews and setting bones—activating blood and unblocking collaterals—tonifying liver and kidney” strategy, guided by traditional pathways, has accumulated rich clinical experience, and its efficacy is undisputed. Building upon this foundation, this model expands new intervention dimensions: guiding the direction of stem cell homing by modulating chemotactic efficiency, and re-establishing the functional integrity of the bone-muscle interface by regulating the differentiation balance. This provides an operational molecular framework for TCM to transition from empirical medication to precision regulation targeting the stem cell niche.

## 6 Implications of the theoretical reinterpretation for TCM orthopedics and traumatology treatment principles and research paradigms

We acknowledge that the connections between the DSCD model and TCM principles, particularly “tonifying the liver and kidney,” remain speculative. The following discussion offers a theoretical framework for future investigation rather than established conclusions.

Treatment Principle Optimization: From Empirical Synergy to Target Synergy. The principle of “treating sinew and bone with equal emphasis” mandates synchronous management of bone and sinew injuries during fracture treatment, and its clinical efficacy has been quantitatively validated in conditions like ankle fractures. This model offers a potential cellular-level explanation for this principle: the repair of bone and sinew may share the same stem cell communication network—SDF-1 released from the fracture end not only recruits BMSCs to initiate the osteogenic program but also, via the circulatory system, might attract MuSCs to participate in muscle fiber reconstruction; conversely, DAMPs released from injured sinew tissue could also mobilize BMSCs to home to the injury site. Accordingly, the biological essence of “treating sinew and bone simultaneously” could be hypothesized as the synergistic activation of the chemotaxis and differentiation programs of dual stem cells, thereby accelerating the functional reconstruction of the bone-muscle composite interface. This understanding advances the design of clinical treatment principles from the empirical consensus of “synchronously handling sinew and bone” to the target synergy level of “regulating the SDF-1/CXCR4 chemotactic axis to guide bidirectional homing, and regulating the Wnt/BMP differentiation balance to coordinate osteogenesis and myogenesis,” providing clear molecular targets for precision intervention.

TCM Mechanism Reinterpretation: From Visceral Regulation to Niche Intervention. The pharmacological basis of the traditional therapeutic method of “tonifying the liver and kidney, strengthening sinews and bones” is often explained as indirectly acting on the sinew-bone system by regulating liver and kidney functions. We propose a testable hypothesis that the active components of liver-kidney-tonifying Chinese herbs may directly act on multiple nodes of the dual stem cell niche. Total flavonoids of Drynaria fortunei activate the Wnt/β-catenin pathway to promote BMSC osteogenic differentiation; Icariin upregulates CXCR4 expression to enhance stem cell homing efficiency; the spleen-invigorating and kidney-tonifying formula improves the chemotactic microenvironment by inhibiting TNF-α—these actions could be uniformly interpreted as systemic interventions on the “stem cell niche”: modulating chemotactic signal intensity to guide homing direction, correcting differentiation pathway deviation to restore the osteogenic-myogenic balance, or remodeling the immune-metabolic state of the microenvironment to maintain niche homeostasis. However, direct experimental evidence linking “tonifying the liver and kidney” to stem cell niche modulation is currently lacking. This represents a key direction for future research. This perspective materializes “tonifying the liver and kidney” from a vague visceral regulation concept into a hypothesized targeted maintenance of the bone-muscle stem cell niche, providing measurable evaluation dimensions for TCM mechanism-of-action research and opening new avenues for formula optimization based on stem cell niche modulation.

Research Paradigm Inspiration: Establishing a Testable Framework for “Sinew Injury Recruiting Bone, Bone Damage Summoning Muscle.” The verifiability of traditional sinew-bone theory has long been constrained, with the crux being the difficulty in translating its core propositions into operational experimental hypotheses. This model decomposes the core proposition of “sinew-bone interdependence” into a set of testable questions: (1) After muscle injury, can MuSC-derived chemotactic signals be confirmed *in vivo* to recruit BMSCs to the muscle injury site? (2) In states of osteoporosis or fracture, can BMSC-derived exosomes be tracked to skeletal muscle and confirmed to activate MuSC proliferation and differentiation programs? (3) Can TCM intervention improve the repair quality and functional recovery of the bone-muscle composite unit by modulating the aforementioned chemotactic or differentiation nodes? These questions propel sinew-bone theory research from literature textual criticism and clinical experience summarization to modern experimental frameworks such as lineage tracing, single-cell multi-omics, and conditional genetic modification, rendering the scientific validation of traditional theory an executable plan. The experimental models, key detection techniques, expected results, and falsification conditions corresponding to the three hypotheses are systematically listed in Table 3.

**TABLE 3:** Testable propositions and experimental validation strategies for core hypotheses of the DSCD model.

Hypothesis no.	Core hypothesis	Suggested experimental model	Key detection techniques	Expected results	Falsification conditions
H1	Following muscle injury, MuSC-derived chemotactic signals can recruit BMSCs to the muscle injury site under *in vivo* conditions	Tibialis anterior (TA) freeze injury/Cardiotoxin (CTX) injection model	MuSC-specific Cre-driven lineage tracing + BMSC *in vivo* two-photon imaging + SDF-1 conditional knockout	After muscle injury, distal BMSCs migrate directionally to the injury site; migration efficiency positively correlates with local SDF-1 concentration; migration is significantly attenuated upon SDF-1 knockout	Lineage tracing fails to detect cross-tissue migration signals from BMSCs, or the number of migrating cells shows no significant difference compared to uninjured controls
H2	In osteoporotic or fracture states, BMSC-derived exosomes can be tracked to skeletal muscle and activate MuSC proliferation and differentiation programs	OVX osteoporosis model/Femoral fracture model	BMSC-specific exosome fluorescent labeling (e.g., CD63-GFP) + MuSC proliferation marker (Pax7^+^/Ki67^+^) immunofluorescence co-staining + MyoD/Myogenin expression detection	Labeled exosomes enrich in skeletal muscle tissue; local MuSC proliferation rate significantly increases; MyoD expression is upregulated	Labeled exosomes are not taken up by skeletal muscle tissue, or MuSC proliferation rate and MyoD expression after uptake show no significant difference compared to controls
H3	TCM intervention can improve the repair quality of the bone-muscle composite unit by modulating chemotactic or differentiation nodes	Bone-muscle composite injury model (combined fracture + muscle contusion)	Conditional gene knockout (SDF-1 KO, β-catenin KO) + TCM monomer/formula intervention + μCT bone morphometry + muscle fiber cross-sectional area and contractility testing	The bone-muscle interface repair quality (bone mass, muscle strength) in the TCM intervention group is significantly superior to the model group; the TCM effect is weakened or disappears upon knockout of key nodes	TCM intervention still exerts a full repair effect after conditional knockout of SDF-1 or β-catenin (indicating the effect is mediated via non-presumed pathways)

H1-H3 correspond to the three core hypotheses: “sinew injury recruiting bone” (chemotaxis), “bone damage summoning muscle” (exosome communication), and “TCM, niche targeting,” respectively. For each hypothesis, suggested experimental models, key detection techniques, expected results, and explicit falsification conditions are provided, reflecting the operationalizability and falsifiability of the theoretical model. CTX, cardiotoxin; OVX, ovariectomy; TA, tibialis anterior; μCT, Micro-computed tomography.

## 7 Limitations and considerations

Several limitations of the proposed DSCD model must be acknowledged. First, as emphasized throughout this article, direct *in vivo* evidence for bidirectional chemotaxis between endogenous MuSCs and BMSCs is currently lacking. Most existing studies have examined general mesenchymal stem cell homing or indirect muscle-bone crosstalk using exogenous cell transplantation models. Whether endogenous MuSCs and BMSCs naturally exhibit reciprocal migration under physiological or pathological conditions remains an open question that requires lineage tracing and intravital imaging studies.

Second, the extent of cross-tissue differentiation potential between MuSCs and BMSCs is not fully defined. While *in vitro* studies suggest lineage plasticity, the degree to which these stem cells can adopt alternative fates in vivo—particularly across the bone-muscle boundary—requires rigorous clonal analysis and lineage tracing.

Third, translational barriers exist between rodent models and human patients. Species differences in stem cell biology, microenvironmental composition, and regenerative capacity may affect the generalizability of findings from animal models to clinical TCM applications.

Fourth, the multi-target nature of TCM compounds, while advantageous, complicates mechanistic dissection. Establishing causal relationships between specific TCM components, stem cell niche modulation, and therapeutic outcomes will require systematic approaches including conditional gene knockout and chemical biology strategies.

Fifth, the DSCD model is intentionally reductive, focusing on two stem cell populations and a limited set of signaling pathways. The complex interplay with immune cells, vascular cells, and neural components of the stem cell niche is not fully captured and warrants future integration.

## 8 Conclusion and outlook

This paper, using the bidirectional chemotaxis and lineage plasticity of skeletal muscle satellite cells and bone marrow mesenchymal stem cells as a thread, has undertaken a systematic cellular-molecular reinterpretation of the TCM “sinew-bone interdependence” theory, forming the following core conclusions.

First, the physiological synergy and pathological inter-transmission between sinew and bone may possess a cellular basis constituted by dual stem cells. Through the synergistic regulation of the SDF-1/CXCR4 chemotactic axis, exosome communication networks, and signaling pathways such as Wnt/BMP/Notch, MuSCs and BMSCs might achieve bidirectional chemotaxis and functional complementarity. “Sinew injury recruiting bone, bone damage summoning muscle” thereby gains a hypothesized mechanistic explanation at the molecular level, pending direct experimental validation.

Second, the scientific connotations of traditional treatment principles such as “treating sinew and bone with equal emphasis” and “tonifying the liver and kidney” can be partially reinterpreted as chemotactic guidance and differentiation regulation of the bone-muscle stem cell niche—offering a testable framework. The multi-target action characteristics of TCM compound formulas and monomers precisely align with the intrinsic need for multi-nodal intervention on this niche, providing a theoretical interface for TCM to transition from empirical medication to targeted regulation.

Third, the traditional interpretive pathways and the model presented here form a complementary rather than substitutive relationship. The former provides a macroscopic functional framework and clinical wisdom from anatomical, visceral manifestation, and biomechanical dimensions, while the latter injects operational logic and actionable intervention nodes at the cellular and molecular levels. Together, they constitute a modern explanatory system for the “sinew-bone interdependence” theory, enabling it to maintain its holistic view advantages while acquiring the capacity for deep integration with modern biomedicine.

Looking ahead, three directions warrant prioritized advancement. First, direct *in vivo* evidence for the reciprocal chemotaxis of skeletal-muscle dual stem cells remains relatively weak. It is necessary to utilize techniques such as dual-recombinase lineage tracing and intravital two-photon imaging to dynamically capture the migration trajectories and interaction timelines of the two stem cell types in bone-muscle composite injury models, thereby clarifying the spatiotemporal kinetic characteristics of “sinew injury recruiting bone, bone damage summoning muscle.” Second, research on the concurrent regulation of bidirectional differentiation during bone-muscle interface regeneration urgently needs to be undertaken. Whether a single stem cell can simultaneously contribute progeny cells in both osteogenic and myogenic directions, or whether the two stem cell types achieve lineage complementarity through exosome-mediated exchange of differentiation commands, awaits elucidation through single-cell multi-omics and spatial transcriptomics. Third, the “niche targeting” hypothesis of TCM intervention requires systematic validation. Conditional gene knockout should be employed to establish the necessity of key chemotactic factors or signaling nodes. Bone-muscle composite injury models should be established, followed by intervention with TCM monomers or formulas to observe whether the repair effect is mediated through these nodes, thereby achieving a mechanistic leap from “TCM is effective” to “how TCM is effective.”

Ultimately, through this theoretical reconstruction, this paper aspires to inject measurable, verifiable, and translatable molecular connotations into the classical theories of TCM orthopedics and traumatology, propelling their development and clinical translation in the era of regenerative medicine.
